# Conserved, unstructured regions in *Pseudomonas aeruginosa* PilO are important for type IVa pilus function

**DOI:** 10.1038/s41598-018-20925-w

**Published:** 2018-02-08

**Authors:** T. L. Leighton, M. C. Mok, M. S. Junop, P. L. Howell, L. L. Burrows

**Affiliations:** 1 Department of Biochemistry and Biomedical Sciences and the Michael G. DeGroote Institute for Infectious Disease Research, McMaster University, Hamilton, ON Canada; 2 Department of Biochemistry, Western University, London, ON Canada; 3 Program in Molecular Structure & Function, The Hospital for Sick Children, Toronto, ON Canada; 4 Department of Biochemistry, University of Toronto, Toronto, ON Canada

## Abstract

*Pseudomonas aeruginosa* uses long, thin fibres called type IV pili (T4P) for adherence to surfaces, biofilm formation, and twitching motility. A conserved subcomplex of PilMNOP is required for extension and retraction of T4P. To better understand its function, we attempted to co-crystallize the soluble periplasmic portions of PilNOP, using reductive surface methylation to promote crystal formation. Only PilO_Δ109_ crystallized; its structure was determined to 1.7 Å resolution using molecular replacement. This new structure revealed two novel features: a shorter N-terminal α1-helix followed by a longer unstructured loop, and a discontinuous β-strand in the second αββ motif, mirroring that in the first motif. PISA analysis identified a potential dimer interface with striking similarity to that of the PilO homolog EpsM from the *Vibrio cholerae* type II secretion system. We identified highly conserved residues within predicted unstructured regions in PilO proteins from various *Pseudomonads* and performed site-directed mutagenesis to assess their role in T4P function. R169D and I170A substitutions decreased surface piliation and twitching motility without disrupting PilO homodimer formation. These residues could form important protein-protein interactions with PilN or PilP. This work furthers our understanding of residues critical for T4aP function.

## Introduction

Type IV pili (T4P) are long, thin (5–8 nm diameter) hair-like appendages which extend from the bacterial surface and promote attachment, cell-cell aggregation, biofilm formation, and twitching motility^1–6^. T4P – which include two major subfamilies, T4aP and T4bP, that vary in terms of assembly system components – are produced by a wide variety of bacteria and archaea, including the opportunistic pathogen, *Pseudomonas aeruginosa*. This bacterium, notorious for its resistance to several classes of antibiotics, infects immunocompromised individuals such as those with severe burns, cystic fibrosis, or acquired immune deficiency syndrome (AIDS)^7^. Mutants lacking T4aP are impaired in host colonization and thus less infectious^8,9^. T4aP also mediate twitching motility, a form of flagellum-independent movement caused by repeated rounds of pilus extension, adhesion, and retraction, allowing the bacteria to pull themselves along surfaces^1,10^. The T4aP system in *P. aeruginosa* is composed of four subcomplexes that together form a transenvelope machinery^3^. The outer membrane (OM) secretin is composed of an oligomer of 14 PilQ monomers and their pilotin, PilF, forming a gated pore through which the pilus is extruded^11,12^. The inner membrane (IM) motor subcomplex is composed of a platform protein PilC^13^, and three cytoplasmic ATPases, PilBTU^14^. The secretin and motor subcomplexes are linked by the alignment subcomplex, PilMNOP, with putative roles in control of pilus assembly-disassembly dynamics and gating of the secretin^15–17^. The final subcomplex is the pilus fibre, which extends from the cell^4,18–20^. This fibre is composed primarily of PilA monomers plus small amounts of minor pilins (PilVWXE/FimU) and PilY1^4,19–21^. These subcomplexes – plus several regulatory proteins whose functions are not well understood^22–25^ – form a fully functional T4aP system. PilMNOP are critical for function of the T4aP system^3,4,26^. Cytoplasmic component PilM is structurally similar to the bacterial actin-like cytoskeletal protein, FtsA^27–29^. The structures of *Thermus thermophilus* PilM bound to a PilN N-terminal peptide and a *P. aeruginosa* PilM-PilN_1–12_ chimera were recently determined by X-ray crystallography, revealing a PilM binding pocket that interacts with a highly conserved motif in PilN’s N-terminus^28–30^. Although no structure for PilN from *P. aeruginosa* is yet available, it is predicted to resemble PilO^30^. The structure of PilN from *T. thermophilus* has been determined^31^, but has a different arrangement of secondary structure elements compared to the predicted structure of PilN from *P. aeruginosa*. A crystal structure of an N-terminally truncated (Δ1–68) version of a PilO dimer was previously determined (PDB 2RJZ)^30^, and the observed homodimer interface later shown to be physiologically relevant^32^. PilN and PilO also form heterodimers^15,17,30,33^. They are predicted to have similar topologies, with short cytoplasmic N-termini preceding single transmembrane segments (TMS), followed by periplasmic segments consisting of a coiled-coiled domain linked to a core domain containing two ferredoxin-like αββ motifs^30^. Although PilN and PilO form both homo- and heterodimers *in vivo*^32^, the IM-associated lipoprotein PilP binds only PilNO heterodimers through its unstructured N-terminal region^17^. Finally, the C-terminal β-domain of PilP interacts with the secretin monomer, PilQ^16^. These protein-protein interactions form a continuous network through the periplasm, as confirmed by a recent 4 nm cryoelectron tomographic model of the *Myxococcus xanthus* T4aP system^34^.

To better characterize physical interactions between alignment subcomplex components, we took a structural approach. Previously, we showed that soluble periplasmic fragments of PilNOP form a stable heterotrimeric complex *in vitro*^17^. During a series of systematic attempts to crystallize this stable heterotrimer, we used reductive methylation to promote crystal formation^35^. Although the resulting crystals diffracted to 1.7Å, they were ultimately found to contain only one member of this subcomplex, PilO. The structure of the PilO_Δ109_ monomer was solved by molecular replacement using our previous 2.2Å PilO_Δ68_ structure (PDB 2RJZ) as a template^30^, revealing novel features, including a shorter N-terminal α1-helix and an additional discontinuous β-strand. We identified highly conserved residues that mapped to largely unstructured regions on the PilO structure, and investigated their roles in T4aP function through mutagenesis and phenotypic assays. Alteration of two of the highly conserved residues resulted in reduced piliation and motility, potentially by perturbation of PilO interactions with itself or other partners. These data lend further insight into the function of this key alignment subcomplex component.

## Results

### A new 1.7 Å crystal structure of PilO_Δ109_ reveals novel secondary structure features

**Figure 1:**
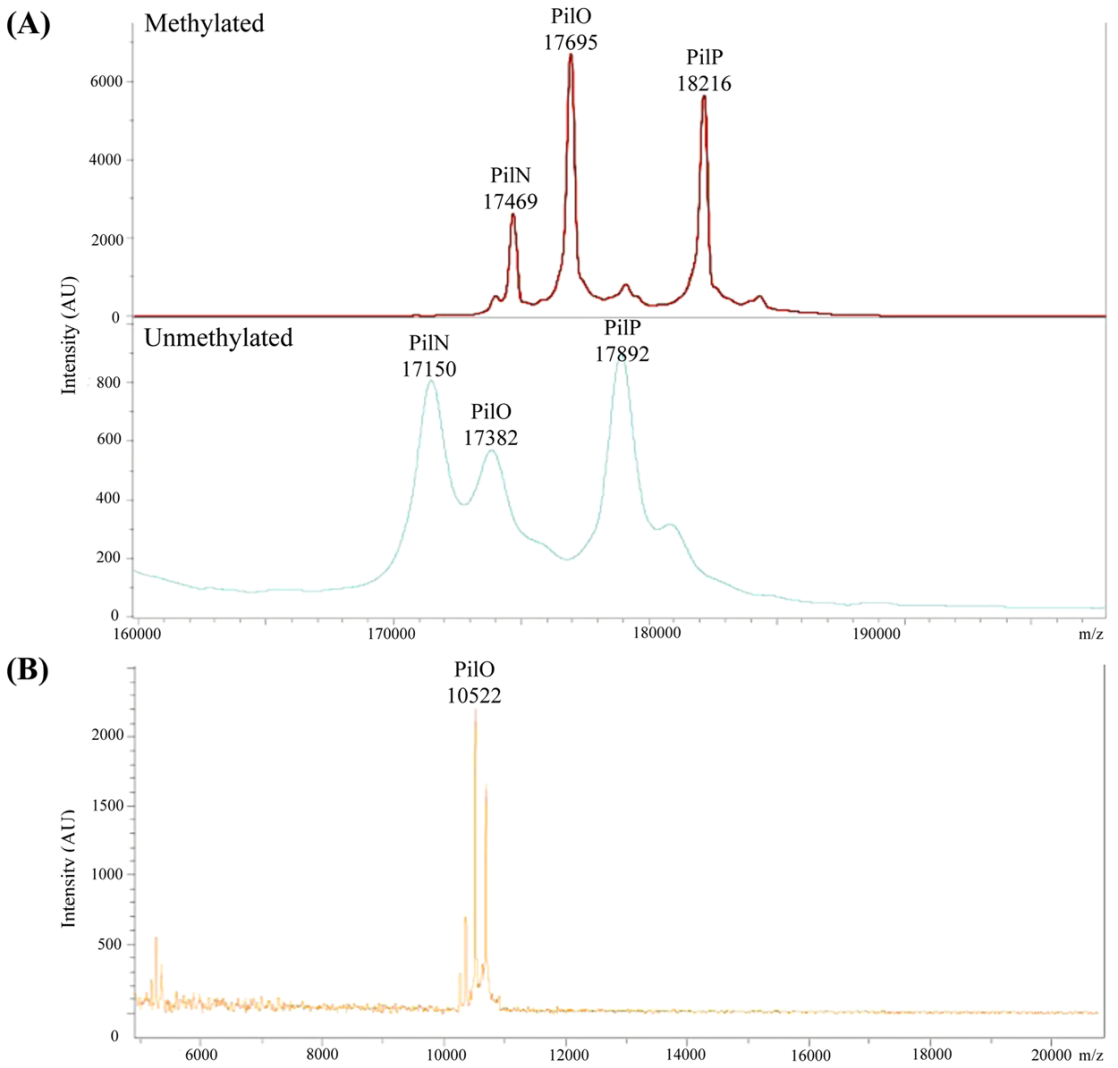
Mass spectrometry (MALDI-TOF/TOF) results indicate successful methylation of the PilN_Δ44_/PilO_Δ51_/PilP_Δ18_ complex but only PilO fragments were present in the crystals. (**A**) Methylated (top) and unmethylated (bottom) samples of 1 mg/mL PilN_Δ44_/PilO_Δ51_/PilP_Δ18_ was analyzed on a Bruker UltrafleXtreme linear detector in positive ion mode. The change in the molecular weight of PilN, PilO and PilP between the methylated and unmethylated samples were 319 Da, 313 Da, and 324 Da, respectively. All three protein fragments have 11 lysine residues, which could be methylated. A single dimethyl-Lys group adds 28 Da. (**B**) Two crystals were washed and resuspended in buffer and was analyzed on a Bruker UltrafleXtreme linear detector in positive ion mode. This weight corresponds to a 110–206 fragment of PilO (approximate molecular weight of 10,560 Da). Remaining peaks–were confirmed to be various PilO fragments. The numbers above the peaks are the molecular weights shown in Da. The chromatogram shows the ion intensities (in arbitrary units) according to mass-to-charge (m/z) ratio.

**Figure 2:**
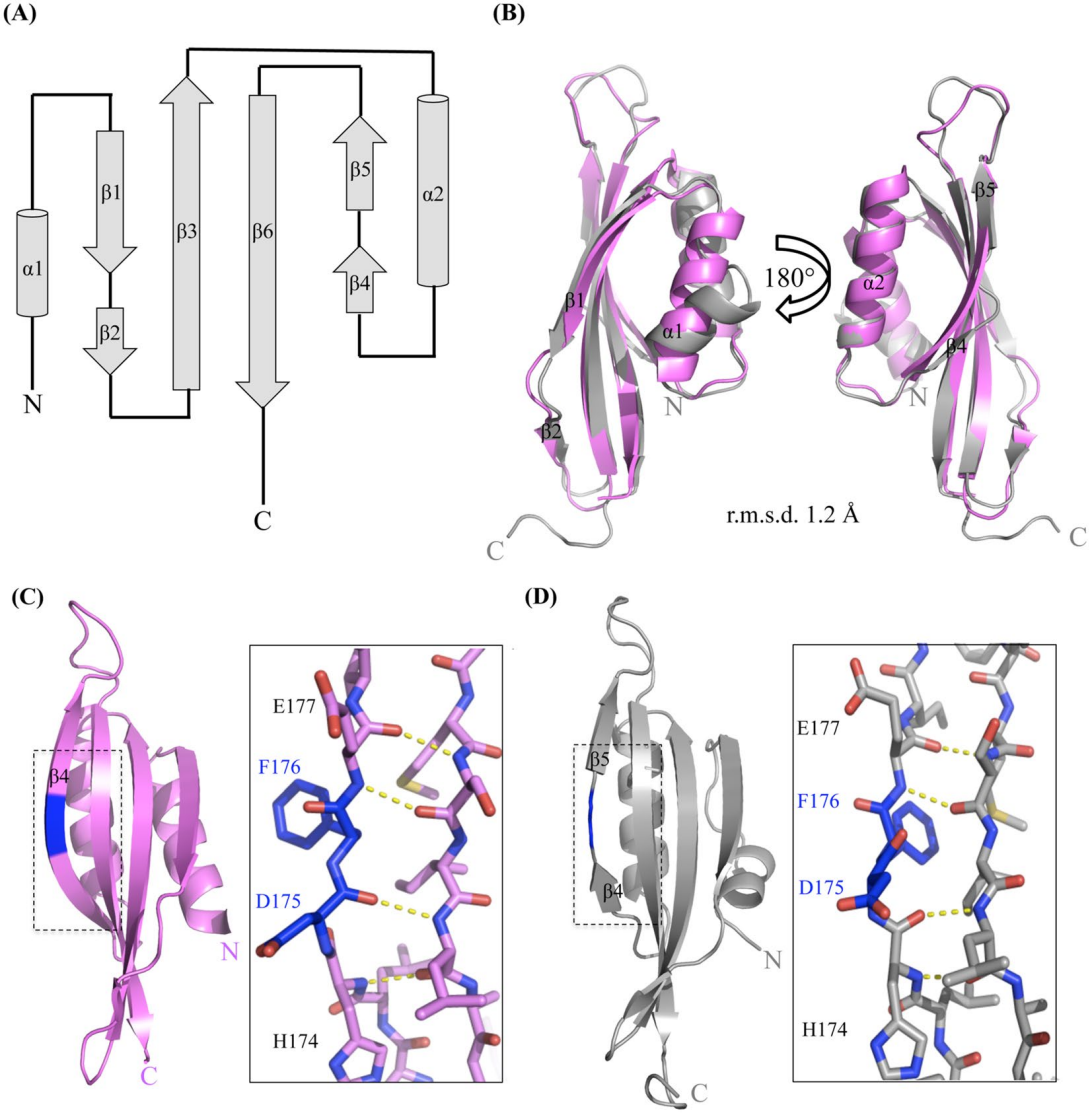
Comparison of *P. aeruginosa* PilO structures. **(A)** Topological diagram of PilO_Δ109_ mapping the N- (P110) and C-termini (K206). Helices α1 and α2 are 8 and 13 residues in length, respectively, while β3 and β6 are 12 residues long. Beta strands β1 and β2 are 8 and 3 residues in length, whereas β4 and β5 have 3 and 4 residues, respectively. **(B)** Comparison of the PilO_Δ109_ structure (grey; PDB 5UVR) with the equivalent residues (110–206) of PilO^2RJZ^_Δ109_ (violet; PDB 2RJZ^30^). **(C)** Reverse view of the PilO^2RJZ^_Δ109_ structure (violet), highlighting the β4 strand residues, D175 and F176 (blue), participating in hydrogen bonding with the β5 strand. **(D)** The equivalent region of the PilO_Δ109_ structure (grey) highlighting the second discontinuous β4β5-strand (inset) and the D175 and F176 residues (blue) not participating in hydrogen bonding with the β6 strand. Hydrogen bonding is indicated by the yellow dashed lines.

To better understand how PilN and PilO interact with one another and with PilP, we attempted to solve the structure of a stable, soluble heterotrimeric complex of PilN_Δ44_/PilO_Δ51_/PilP_Δ18_^17^_._ Although the protein complex was soluble, abundant, and stable over long periods of time at various temperatures, crystallization was unsuccessful. To promote crystallization we explored the use of reductive methylation, thought to promote crystal formation through chemical modification of surface-exposed lysines to decrease surface entropy^35^. All three proteins were present after reductive methylation as determined by SDS-PAGE and Coomassie staining, and methylation was confirmed by matrix-assisted laser desorption/ionization time-of-flight/time-of-flight mass spectrometry (MALDI-TOF/TOF MS) (Fig. 1A). A small array of hexagonal pyramid shaped crystals were grown and data were collected at the Canadian Light Source (CLS) and processed using Imosfilm^36^. During the preliminary analysis of the data, it became evident that only one of the three proteins (PilO) was present in the crystal. Although the PilO construct used encompassed residues 52–208, we could model only residues 110–206. Analysis of the crystals using MALDI-TOF/TOF MS confirmed that proteolysis had occurred during the crystallization process (Fig. 1B). Various PilO fragments were present, with the largest peak corresponding to a PilO fragment with a molecular weight of 10,522 Da, similar to the predicted mass of 10,560 Da for a PilO 110–206 fragment (Fig. 1B). Using our previous 2.2 Å PilO_Δ68_ structure (PDB 2RJZ)^30^ as a template, a 1.7 Å structure of PilO_Δ109_ was solved by molecular replacement and refined to an R_work_/R_free_ value of 23.1/26.7% (Table 1 and Fig. 2).The new structure (PDB 5UVR) encompasses residues P110 to K206, equivalent to ~60% of the periplasmic region (just under 50% of the total protein). PilO_Δ109_ has two αββ motifs composed of α1-β1β2-β3 and α2-β4β5-β6 (Fig. 2A). The four β-strands form an antiparallel β-sheet onto which the two α-helices pack. A pseudo-2-fold axis within the β-sheet relates the two compact αββ-subdomains with a root mean square deviation (r.m.s.d.) of 3.4 Å for 47 Cα atoms. The 2-residue linker between β1β2 includes P134 and E135, while the 2-residue linker between β4β5 contains D175 and F176; these residues are responsible for the observed strand discontinuity. To directly compare the new and previous structures, we truncated the latter to include only residues 110–206 (known henceforth as PilO^2RJZ^_Δ109_). The two structures have an r.m.s.d. of 1.2 Å over 97 Cα backbone atoms (Fig. 2B). Compared to the previous structure, the N-terminal α1-helix in PilO_Δ109_ is shorter, with more of the surrounding region unstructured and tilted ~45° away from the β-sheet, which could be due to the loss of the N-terminal coiled-coil regions in the PilO^2^ structure, or to differences in the crystallization conditions (Fig. 2B – left). A second discontinuous β-strand (β4β5) – not present in the PilO^2RJZ^_Δ109_ structure – was identified in the new structure (Fig. 2B – right). The main chain carbonyls of D175 and F176 do not participate in hydrogen bonding between the β4β5- and β6-strands in the PilO_Δ109_ structure, creating the discontinuous β-strand (Fig. 2D). In the previous PilO structure, hydrogen bonding was complete through this segment (Fig. 2C).

**Table 1:** Data collection and refinement statistics for PilO_Δ109_.

Data Collection	
Beamline	CLS 08ID-1
Wavelength (Å)	0.979
Space group	*P*6_1_22
*a*, *b*, *c* (Å)	40.8, 40.8, 250.5
*α*, *β*, *γ* (°)	90, 90, 120
Resolution range (Å)	35.34-1.7 (1.79-1.7)
Total reflections	263281
Unique reflections	14759
Redundancy	6.7 (6.9)
Completeness (%)	99.9 (100)
Mean *I*/*I)*	10.8 (2.9)
*R*_merge_ (%)	7.8 (54.2)
Anisotropic deltaB	11.52
Mosaicity (°)	0.54
**Structure Refinement**	
*R*_work/free_ (%)^*^	23.1/26.7
R.m.s.d. Bond lengths (Å)	0.005
R.m.s.d. Bond angles (°)	0.769
Ramachandran plot^‡^	
Total favoured (%)	98
Total allowed (%)	2
Coordinate error (Å)^§^	0.14
Wilson B factor	23.7
Atoms	
No. protein atoms	849
No. water	97
Average B-factors (Å^2^)^§^	
Protein	40.58
Water	47.70

Note: Values in parentheses correspond to the highest resolution shell. ^*^R_work_ = ∑ | |F_obs_| − k|F_calc_| |/|F_obs_| where F_obs_ and F_calc_ are the observed and calculated structure factors, respectively. R_free_ is the sum extended over a subset of reflections (5%) excluded from all stages of the refinement. ^‡^As calculated using MolProbity^49^. ^§^Maximum-likelihood based Coordinate Error and Average B-factors, as determined by PHENIX^42^

### The interface for the predicted PilO_Δ109_ dimer is similar to that of EspM

**Figure 3:**
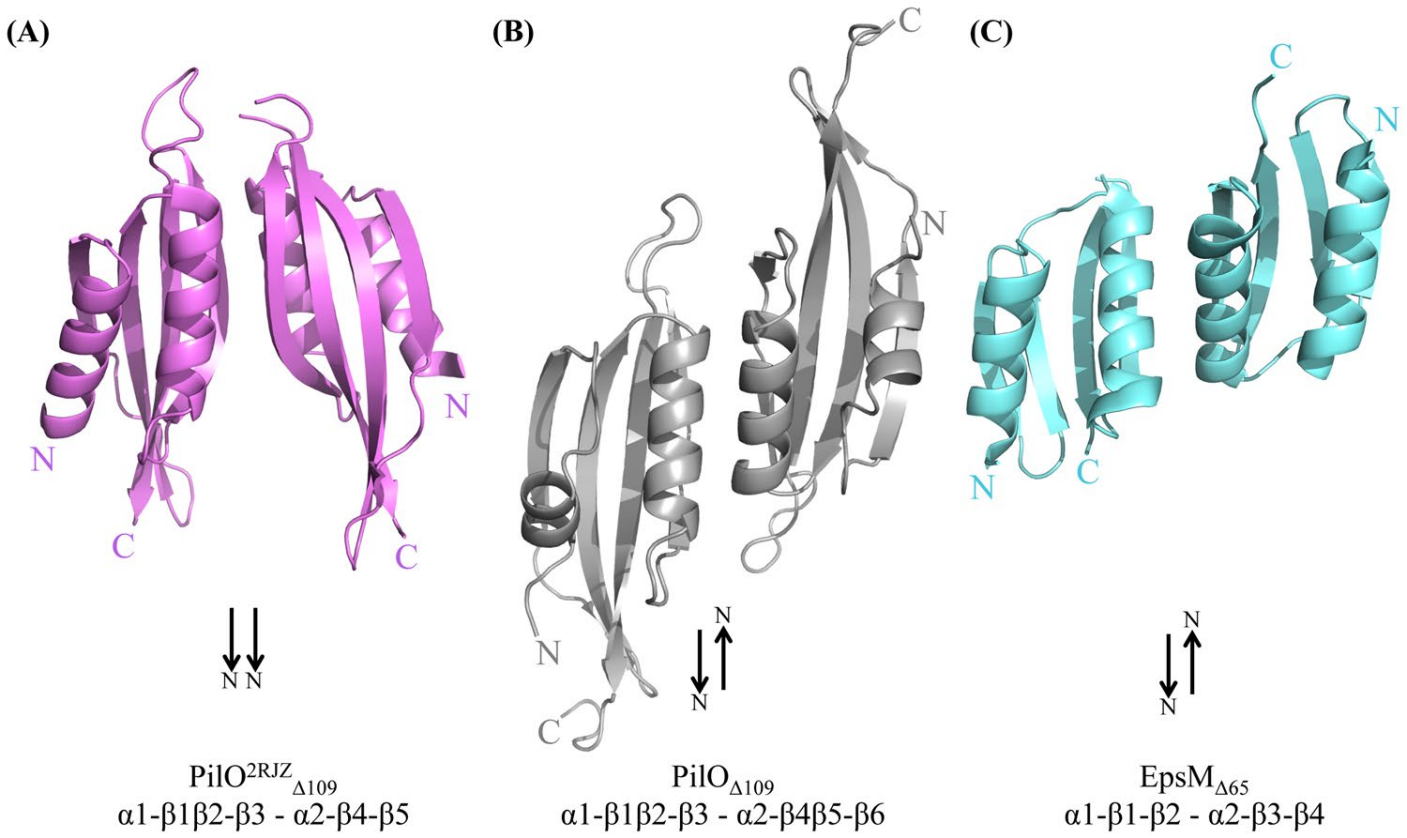
The interface of the predicted PilO_Δ109_ dimer is similar to that of EpsM. **(A**) The dimer interface found in the PilO^2RJZ^_Δ109_ dimer (violet) (PDB 2RJZ^30^). **(B)** The most energetically favourable predicted interaction interface for the PilO_Δ109_ structure (grey). **(C)** The EpsM_Δ65_ crystal structure (cyan) from the T2SS of *V. cholerae* (PDB 1UV7^38^). Black arrows indicate direction of the N-termini for each subunit.

One molecule of PilO _Δ109_ was found in the asymmetric unit. Using PISA software^37^, two possible interaction interfaces with crystallographic symmetry mates were identified, based on the protein-protein interactions in the crystal lattice. These interfaces bury 1,180 and 1,140 Å^2^, respectively, compared with a total surface area of approximately 11,500 Å^2^. The dimer with the largest buried surface area, considered to be more energetically favourable (-12 kcal/mol), was analyzed further (Fig. 3B). The interface between the core domains of PilO^2RJZ^_Δ109_ was formed by interactions between the α2-helix of monomer 1 and the β4-strand of monomer 2 (Fig. 3A)^30^. These contacts are similar to the new structure of PilO_Δ109,_ where the α2-helix and β4β5-strands are the main points of contact in the predicted dimerization interface. However due to the orientation of the individual monomers, the α2-helix of monomer 1 would interact with the α2-helix of monomer 2; similarly, the broken β4β5 strands of each monomer would interact. The monomer orientation in the PilO_Δ109_ dimer places the β-sheets on the same side, forming a longer 8-stranded anti-parallel β-sheet (Fig. 3B). This is in contrast to the PilO^2RJZ^_Δ109_ dimer where the β-sheets and α-helices are on opposite sides (Fig. 3A). The PilO_Δ109_ interface resembles that reported for the 1.7 Å resolution structure of its homologue EpsM_Δ65_ from the *V. cholerae* T2SS, with the monomers arranged in the same antiparallel orientation (Fig. 3C)(PDB 1UV7^38^). EpsM_Δ65_ has dimerization contacts between the α2-helices of each monomer and between the β3-strands, equivalent to β4β5 in the PilO_Δ109_ structure (Fig. 3B)^38^. This organization orients the N- and C-termini of the individual PilO_Δ109_ and EpsM_Δ65_ monomers in opposite directions, compared to the PilO^2RJZ^_Δ109_ structure where the N- and C-termini are oriented in the same direction, which we believe to be the more physiologically relevant conformation (Fig. 3A).

### Two highly conserved hydrophobic segments correspond to unstructured loop regions

**Figure 4:**
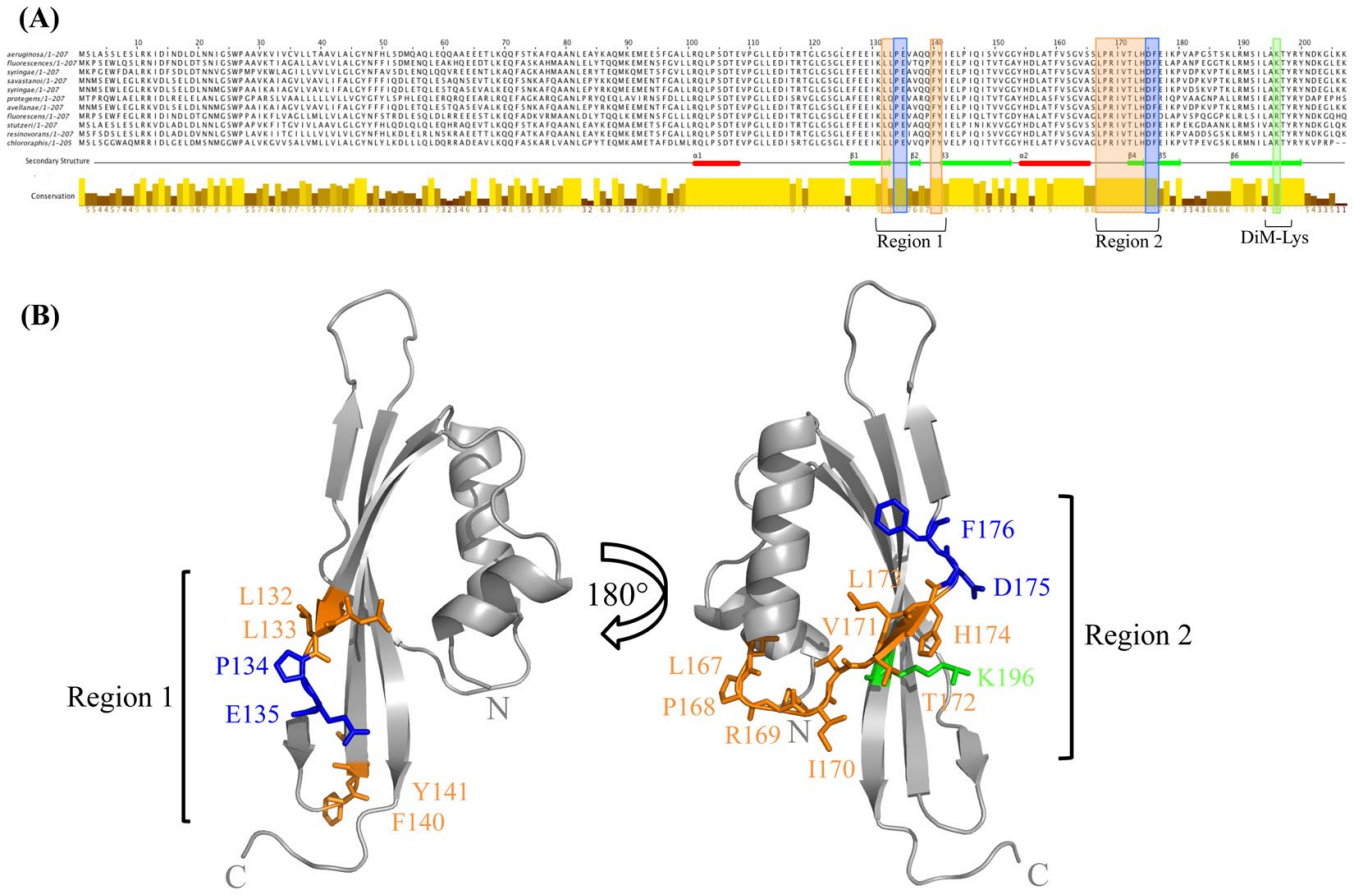
Highly conserved residues in unstructured regions on the PilO_Δ109_ structure probed by site directed mutagenesis. (**A**) The sequence conservation of the PilO families from a subset of Pseudomonads (*P. fluorescens*, *P. syringae pv. phaseolicola* 1448 A, *P. savastanoi, P. syringae, P. protegens* CHA0*, P. avellanae, P. stutzeri* A1501*, P. resinovorans, P. chlororaphis*) are indicated by the bars. The conservation of the residues is indicated by the bar and the intensity of the color (high conservation, high bar and bright yellow; low conservation, low bar and a dark brown color). The α-helices (red rectangles) and β-strands (green arrows) indicate secondary structure elements present in the PilO_Δ109_ structure. **(B)** The structure of PilO_Δ109_ indicating the position of the residues chosen for site directed mutagenesis. Conserved and unstructured residues (orange), β-strand breakers (blue), and the position of the di-methyl Lys (green), are indicated.

The PilMNOP subcomplex is highly conserved in *Pseudomonads* and other T4aP expressing bacteria^6^. Alignment of *P. aeruginosa* PilO with homologues from various Pseudomonads revealed a number of notable features (Fig. 4A). The N-terminal residues (1–99) share relatively limited overall sequence conservation, whereas the core domain, consisting of the two αββ motifs, is more highly conserved, especially in regions of defined secondary structure. The regions corresponding to α1 and α2 are highly conserved, as are the residues immediately following the α1-helix (109–116). In our previous structure, these were part of the longer α1-helix, but in the new PilO_Δ109_ model, they are unstructured (Fig. 2). The β-strands are also relatively conserved, but interestingly, there is a high degree of conservation in two largely hydrophobic regions, corresponding to the unstructured areas surrounding the discontinuous β2β3- and β4β5-strands. In the new structure, a dimethyl-Lys residue (K196) was identified on the back face of the β6-strand (Fig. 4B), the result of the reductive methylation used to promote crystallization of the PilN_Δ44_/PilO_Δ51_/PilP_Δ18_ complex (Fig. 1A). The PilN_Δ44_/PilO_Δ51_/PilP_Δ18_ fragments are each predicted to have 11 lysine residues potentially available for methylation. The difference in size for each protein post-methylation (PilN 319 Da, PilO 313 Da, and PilP 324 Da), suggested that ~10 lysine residues each (28 Da per lysine), plus an additional 28 Da for the N terminus of each protein, were modified. However, only K196 could be modelled, while the remaining lysines (K131, K179, K188, K203, and K206) were missing electron density. Although this meant we could not confirm structurally that these other residues were dimethylated, the mass spectrometry results were consistent with this modification. Region 1 includes residues L132, L133, F140, Y141, plus “β-strand breaker” residues P134 and E135 (Fig. 4B - left). Region 2, on the opposite side of the PilO_Δ109_ model, consists of residues L167, P168, R169, I170, V171, T172, L173, H174, and the β-strand breaker residues D175 and F176 (Fig. 4B - right). The LPRIVTL residues (167–173) in region 2 were previously identified as the most highly conserved segment in PilO^30^. Unstructured regions of proteins can often undergo conformational changes to a more ordered state upon interaction with their target substrates – including other proteins^39^. With this information, we hypothesized that these highly conserved residues found in largely unstructured regions could play a potential role in protein-protein self interactions (homodimers) or with PilN and/or PilP. The residues in this region were mutated (individually or in pairs; Table 2) by introducing point mutations onto the *P. aeruginosa* chromosome at the *pilO* locus to preserve the native stoichiometry and expression levels that are important for T4aP function^33^.

**Table 2:** Summary of PilO mutants and their phenotypes.

PilO	Location	Twitching motility	Surface pili
LL132-133AA	Region 1 – β1	Yes	Yes
PE134-135AL	Region 1 – β1	Yes	Yes
F140A	Region 1 – β2–β3	Yes	Yes
Y141A	Region 1 – β2-β3	Yes	Yes
L167A	Region 2 – α2-β4	Yes	Yes
P168A	Region 2 – α2-β4	Yes	Yes
R169D	Region 2 – α2-β4	Reduced (≈ 40%)	Reduced (≈ 65%)
I170A	Region 2 – α2-β4	Reduced (≈ 50%)	Reduced (≈ 70%)
V171A	Region 2 – α2-β4	Yes	Yes
TL172-173AA	Region 2 – β4	Yes	Yes
H174A	Region 2 – β4	Yes	Yes
D175R	Region 2 – β4-β5	Yes	Yes
F176A	Region 2 – β4-β5	Yes	Yes

**Figure 5:**
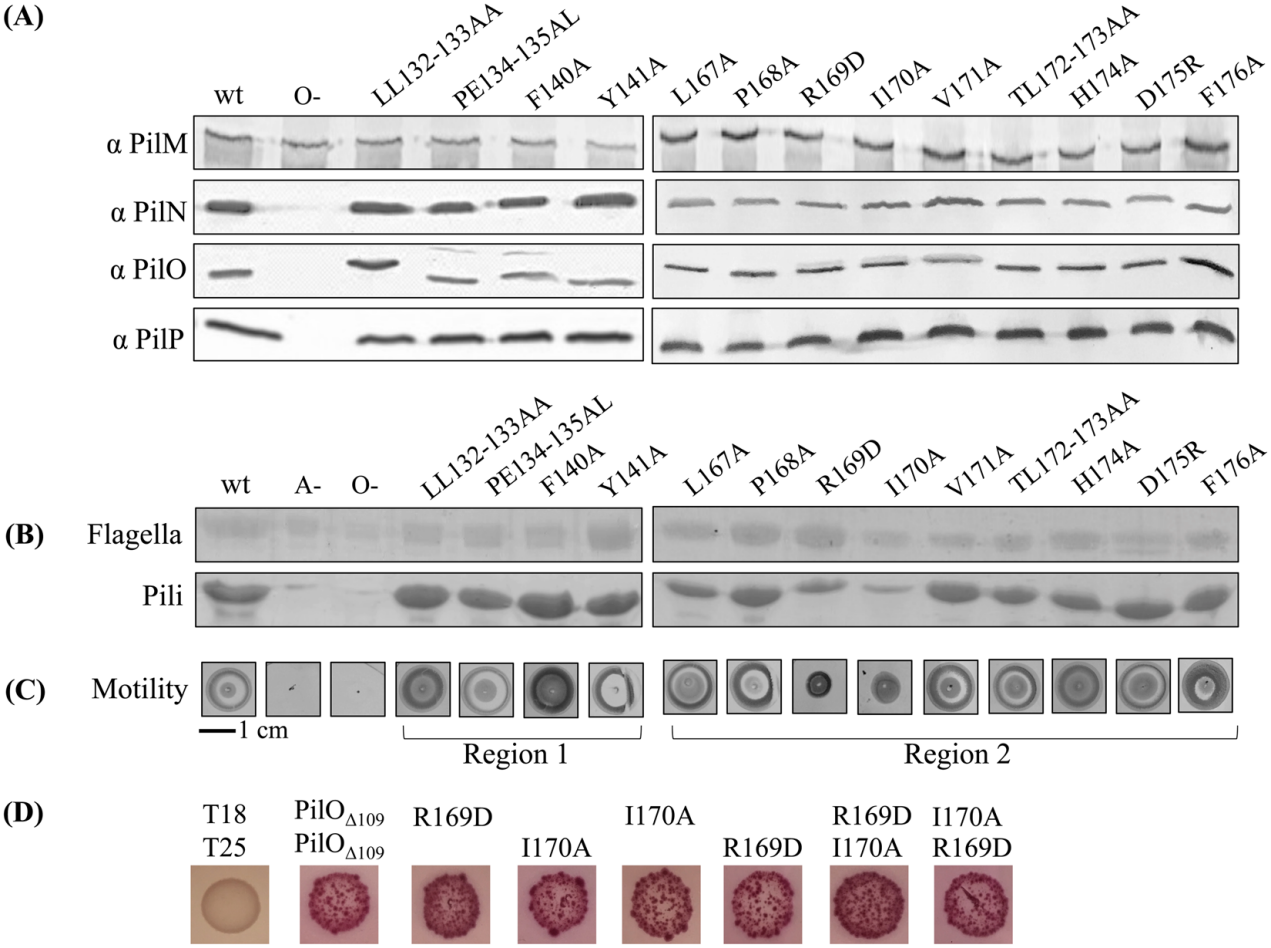
PilO R169D and I170A mutations disrupt T4aP function. All PilO mutant strains, as well as wild-type (wt) and the negative control (O−), were tested for **(A)** expression and stability of alignment subcomplex proteins (PilMNOP) via Western blotting using protein specific antisera as indicated on left, **(B)** sheared surface pili, and **(C)** twitching motility. Sheared surface proteins were separated on 15% SDS-PA gel stained with Coomassie brilliant blue to visualize. For reference, originals of the Western blots and gels are provided in Supplementary Figure S1. Twitching zones were stained with 1% (w/v) crystal violet. A non-piliated strain of *P. aeruginosa* (A-) was included, though some spill over from the wt lane can be detected. **(D)** Interaction between T18-PilO_Δ109_ and T25-PilO_Δ109_ fusion constructs were detected using a bacterial two hybrid assay, on MacConkey agar indicating media supplemented with 1% maltose. Interaction between the core regions of PilO remained despite the presence of either the R169D and/or I170A mutations.

Substitutions designed to disrupt hydrophobic or charged interactions were made (Table 2). Because PilO stability affects that of its protein partners^33^, stable expression of all alignment subcomplex proteins was verified by Western blot using protein-specific antisera (Fig. 5A). Mutants were then assessed for surface pilus expression and twitching motility. Only PilO R169D and I170A reduced twitching motility (approx. 40% and 50% relative to wild-type, respectively) and levels of surface piliation (Figure 5BC). Although lacking the N-terminal residues (68–109) of the previous PilO^2RJZ^ structure, the PilO_Δ109_ core regions interacted in a bacterial two hydrid system^40^. Introduction of either the R169D and/or the I170A substitutions did not disrupt PilO homodimerization (Fig. 5D), suggesting the loss of function could be due to perturbation of interactions with other partners.

## Discussion

Visualizing interfaces among components of the T4aP alignment subcomplex through co-crystallization of a heterotrimeric PilNOP complex has proven to be a challenging goal. Although the soluble, stable complex was used for the crystallization experiments, the resulting crystals contained only a truncated form of PilO (PilO_Δ109_). We suspect that the high pH of CAPS in the crystallization condition, or the prolonged period required for the formation of crystals (6 months), may have led to dissociation of the PilNOP complex, leaving only the core αββ domain of PilO intact. Similar phenomena have been observed for other PilN and PilO homologs, where constructs that initially included the predicted coiled-coils and the αββ core ultimately formed crystals containing only the core region, indicating that it is highly stable to proteolytic degradation^31,38^. Coupling work from our previous study^16^ wherein the N-terminal region of PilP (~18–76) interacts with a PilNO heterodimer, with new information from cryoelectron tomography studies^34^, we now infer that it is mainly the unstructured N-terminal region of PilP interacts with PilNO. Thus, inclusion of the full length PilP protein in the soluble PilNOP complex and mobility of its C-terminal folded domain due to lack of interaction with PilNO may have impeded crystal formation; future work will address this issue.

Though shorter than our previous structure (PDB 2RJZ)^30^, PilO_Δ109_ is of higher resolution and thus provided a new level of structural detail. The αββ fold, a simplified version of the ferredoxin fold (βαβ), was first identified in EpsM from the *Vibrio cholerae* type II secretion system (T2SS), and is typical of PilN and PilO homologues^30,38,41^. Previously, only one discontinuous β-strand was identified in the first αββ motif (Fig. 2C), but the new structure exhibits another β-strand break in the second αββ motif at the same position, resulting in a secondary structure pattern of α1-β1β2-β3 and α2-β4β5-β6 (Fig. 2D). Both discontinuous β-strands are found on the outside edges of the antiparallel β-sheet where they may increase flexibility to accommodate multiple protein-protein interactions, such as recently described PilO homo- and PilNO heterodimers^32^.

Two potential interaction interfaces with crystallographic symmetry mates were investigated^37^. Interestingly, the more energetically favourable interface orients the N- and C-termini of each PilO monomer in opposite directions (Fig. 3B), similar to the interface identified in the T2SS PilO orthologue, EpsM, which crystallized as a homodimer (Fig. 3C) (PDB 1UV7)^38^. For these head-to-tail oriented dimers to be biologically relevant, the PilO and EpsM proteins would have to interact horizontally (parallel to the plane of the membrane rather than vertically as portrayed in Fig. 3B), such that their N- and C-termini would be oriented to the sides to accommodate the membrane anchoring of their transmembrane segments. Instead, it is more likely that the previously observed PilO_Δ68_ dimer interface (Fig. 3A) – in which both N- and C-termini are oriented in the same direction, towards the inner membrane – is the biologically relevant one, similar to the orientation described in a recent cryoelectron tomographic model of the T4aP system of *M. xanthus*^34^.

A large proportion of highly conserved PilO residues are located in regions that lack regular secondary structure (Fig. 4). Of these, only R169D and I170A mutations had effects on piliation and motility (Fig. 5B,C). These residues are found in the unstructured region between the α2-helix and the β4-strand (region 2), previously identified as the most highly conserved motif in PilO orthologues^30^. This region of PilO also participates in both homodimerization and formation of PilNO heterodimers^15,30,32^. These residues cluster near the discontinuous β–strands, a feature not observed in the PilO^2RJZ^_Δ109_ structure (Fig. 2)^30^. The role of these discontinuous β-strands has yet to be determined, as other PilO or PilN homologs appear to have a single, continuous β-strand at the corresponding position^31,38,41^. Split β-strands could afford these regions of PilO more flexibility to accommodate dynamic interactions with other periplasmic T4aP components. However, replacing the “β-strand breaker” residues (P134 and E135 in region 1, or D175 and F176 in region 2) had no effect on T4aP function. PilO homodimerization was unaffected by the R169D and I170A residues as determined using a bacterial two-hybrid assay (Fig. 5D). Whether these residues affect PilO interaction with PilN or PilP is currently under investigation.

In conclusion, we determined a higher-resolution structure of the PilO core domain, revealing new features including a second discontinuous β-strand. Two residues in close proximity to this feature are critical for normal T4aP function. High-resolution structures are useful tools when combined with other structural techniques, such as cryo-electron microscopy or small angle X-ray scattering. For example, atomic structures from the T4aP and T2S systems of various bacterial species were used to model each of the T4aP components in a ~4 nm electron cryo-tomographic envelope of the T4aP system of *M. xanthus*, for which no structures are available^34^. Comparison of models of the piliated and non-piliated states of the T4aP system allowed for insights into the mechanism behind T4aP function^34^. These findings provide a stepping-stone for further investigation of the interactions between the highly conserved alignment subcomplex proteins.

## Methods

### Strains, media and growth conditions

Bacterial strains and plasmids used in this study are listed in Supplementary Table S1. *E. coli* and *P. aeruginosa* were grown at 37 °C in Luria-Bertani (LB) media supplemented with antibiotics at the following final concentrations when necessary (μg/mL): ampicillin (Ap), 100; kanamycin (Kn), 50; gentamicin (Gm), 15 for *E. coli* and 30 for *P. aeruginosa*, unless otherwise specified. Plasmids were transformed by heat shock into chemically competent cells. All constructs were verified by DNA sequencing (MOBIX – McMaster University).

### Expression and purification of PilN_Δ44_/PilO_Δ51_/PilP_Δ18_

N-terminally truncated versions of PilN and PilO were previously cloned into the EcoRI/HindIII and NdeI/XhoI cloning sites, respectively, of a pET28a vector, creating untagged but co-expressed PilN_Δ44_/PilO_Δ51_^17,30^. TOPO cloning was used to introduce an N-terminally truncated form of PilP into a pET151 vector (Invitrogen), creating a PilP_Δ18_ construct with an N-terminal 6-His tag (PilP_Δ18_His_). Based on previous optimization studies, we expressed the PilNO and PilP fragments separately, combining the bacterial pellets at the purification stage. Briefly, the constructs were transformed separately into *E. c*oli BL21 cells and plated on LB agar plates supplemented with either Km (for the pET28a vector) or Ap (for the pET151 vector). A single colony of each transformant was used to inoculate separate 20 mL aliquots of LB containing appropriate antibiotics, and incubated overnight at 37 °C, shaking at 200 rpm. Each overnight culture was used to inoculate 1 L of fresh LB (1:100 dilution) containing antibiotic and the cells were grown at 37 °C, with shaking, to an OD_600_ of approximately 0.6. Protein expression was induced by adding IPTG (isopropyl β-D-1 thiogalactopyranoside, Sigma Aldrich) to the cultures at a final concentration of 1 mM. The cells were incubated for 4 h at 37 °C, prior to being harvested by centrifugation (3,993 × *g*, 15 min, 4 °C) in an Avanti J-26 XPI centrifuge (Beckman Coulter). Bacterial pellets were frozen at −80 °C until further use.

Bacterial pellets were thawed, each resuspended in 10 mL Nickel A buffer (20 mM HEPES pH 7.5, 500 mM KCl, 10 mM imidazole, 10% (v/v) glycerol), then both pellets (PilN_Δ44_/PilO_Δ51_ and PilP_Δ18_His_) were combined into a single 50 mL screw cap tube with 1 complete EDTA-free protease inhibitor tablet (Roche). Cells were lysed via sonication on ice, on setting 4, for 2 min with cycles of 10 s on and 10 s off (Sonicator 3000; Misonix). The lysates were centrifuged (11,000 × *g*, 30 min, 4 °C) in an Avanti J-26 XPI centrifuge (Beckman Coulter) to remove intact cells and other cellular debris. Pelleted material was retained for analysis by SDS-PAGE, while supernatants were filtered through 0.22 μm Acrodisc syringe filter (Pall Corporation). The lysate was purified using Nickel-NTA affinity chromatography on an AKTA start FPLC (VWR). Protein lysate was flowed through a 1 mL His-Trap^TM^ FF column (GE Healthcare) pre-equilibrated with buffer, and washed with Nickel A buffer, then increasing amounts of Nickel B buffer (20 mM HEPES pH 7.5, 500 mM KCl, 300 mM imidizole, 10% (v/v) glycerol) in a linear gradient. The bound protein was eluted from the column in pure Nickel B buffer and collected in 1 mL fractions. An aliquot of each fraction was mixed 1:1 with 2 × reducing or non-reducing SDS-PAGE loading and electrophoresed on a 15% SDS-PA gel. The gel was stained using Coomassie Blue Staining solution (0.1% (w/v) Coomassie Brilliant Blue R-250, 50% (v/v) methanol and 10% (v/v) glacial acetic acid), or developed by Western blot using protein-specific antisera as described below. His-tagged PilP_Δ18_ was successfully able to pull out untagged PilN_Δ44_ and PilO_Δ51_ as previously described^17^.

### TEV Digestion

The PilN_Δ44_/PilO_Δ51_/PilP_Δ18_His_ protein complex elution fraction, plus 500 μL of 1 mg/mL of Tobacco Etch Virus (TEV) protease were added to a 12–30 mL Slide-A-Lyzer Dialysis Cassettes 10 K MWCO (Thermo Scientific). The cassette and its contents were dialyzed into a new buffer (20 mM HEPES pH 7.5, 120 mM NaCl) overnight at 4 °C, stirring. The sample was extracted from the cassette and run through a 1 mL His-Trap^TM^ FF column (GE Healthcare) pre-equilibrated with the dialysis buffer to separate PilN_Δ44_/PilO_Δ51_/PilP_Δ18_ complex from His-tag. Flow through from the column was collected and the protein complex was concentrated down using a Vivaspin-20 10 kDa centrifugal concentrator (GE Healthcare) in an Allegra X-14 (Beckman Coulter) benchtop centrifuge (3,000 × *g*, 30 min, 4 °C) to concentrate the protein complex. A Bradford assay was used to measure the final protein concentration of the complex at approximately 10 mg/mL.

### Reductive Methylation

Modification of surface-exposed lysine residues on the PilN_Δ44_/PilO_Δ51_/PilP_Δ18_ complex was carried out using the Hampton Research Reductive Alkylation kit protocol following manufacturer’s instructions (Hampton Research). Briefly, 20 μL of 1 M dimethylamine borane complex (ABC) was added to 1 mL of 10 mg/mL protein, and inverted to mix. Then 40 μL of 1 M formaldehyde was added to the tube, and incubated for 2 h at 4 °C rocking. After the 2 h incubation, another 20 μL of ABC was added to the tube followed by another 40 μL of 1 M formaldehyde. The tube was incubated again at 4 °C for 2 h. Finally 10 μL of ABC was added to the tube and the reaction incubated overnight at 4 °C with rocking. The reaction was stopped with the addition of 125 μL of 1 M glycine, and incubated for 2 h, rocking at 4 °C. The methylated protein complex was then separated from the reaction products through size exclusion chromatography.

### Size exclusion chromatography of the PilN_Δ44_/PilO_Δ51_/PilP_Δ18_ subcomplex

Analytical gel filtration of the methylated PilN_Δ44_/PilO_Δ51_/PilP_Δ18_ protein complex was performed using an AKTA FPLC (GE Healthcare) equipped with a Superdex S75 10/300 GL (GE Healthcare) column. The column was pre-equilibrated with a 20 mM HEPES pH 7.5 and 120 mM NaCl buffer prior to injection of the protein sample. Typically, 500 μL of a 10 mg/mL protein solution was loaded onto the column. Gel filtrations were run at a flow rate of 0.6 mL/min onto the S75 column at 4 °C, and fractions were collected in a 96-well plate format, with the absorbance at 280 nm monitored over the course of the experiment. The purest PilN_Δ44_/PilO_Δ51_/PilP_Δ18_ protein fractions, as determined by SDS-PAGE and staining with Coomassie blue, were pooled and concentrated down using a Vivaspin 2 (10 kDa) centrifugal concentrator (GE Healthcare) in an Allegra X-14 (Beckman Coulter) benchtop centrifuge (3,000 × *g*, 30 min, 4 °C).

### Crystallization and structural determination

Native PilN_Δ44_/PilO_Δ51_/PilP_Δ18_ crystals were grown using the hanging drop vapour diffusion method in a 1:1 ratio of protein (12 mg/mL in 20 mM HEPES pH 7.5 and 120 mM NaCl) to precipitant (0.2 M NaCl, 0.1 M N-cyclohexyl-3-aminopropanesulfonic acid (CAPS) pH 10.5, 20% (v/v) polyethylene glycol (PEG) 8000) with the addition of 0.2 μL of 30% (v/v) dimethyl sulfoxide (DMSO)) over 1.5 M ammonium sulphate, and kept at 4 °C for approximately 6 months, with trays being checked each week for the first 2 months, then on a monthly basis. Data was collected at 0.979 Å on the 08ID-1 beamline at the Canadian Light Source (CLS) in Saskatchewan, Canada. Data was processed using IMosflm^36^ and the space group was determined to be *P*6_1_22. The structure of PilO was determined by molecular replacement with Phaser-MR from Phenix^42^ using a truncated model of PilO_Δ68_ (PDB 2RJZ^30^) as the search model. Iterative rounds of model building and refinement was carried out using Coot^43^ and Phenix-Refine^42^. During the structure solution, it became clear that only one protein (PilO) was present in the crystal. The PilO protein could be modelled from residue 110 to the C terminus (PilO_Δ109_). The data collection and model refinement statistics are presented in Table 1. Root-mean-square deviation (r.m.s.d) values between the superimposed structures, and graphical presentation were both performed in PyMOL (v 1.8; Schrodinger).

### Mass Spectrometry of the PilN_Δ44_/PilO_Δ51_/PilP_Δ18_ complex

Methylated and unmethylated samples of 1 mg/mLPilN_Δ44_/PilO_Δ51_/PilP_Δ18_ in a 20 mM HEPES pH 7.5 and 120 mM NaCl buffer were sent to the Biointerfaces Institute (McMaster University) for analysis. Briefly, the sample was mixed in a 1:1 ratio with a saturated solution of sinapinic acid prepared in a 30:70 (v/v) Acetonitrile:TFA 0.1% in water. One μL of the samples was spotted on a MALDI pad and subject to matrix-assisted laser desorption/ionization time-of-flight/time-of-flight (MALDI-TOF/TOF) on a Bruker UltrafleXtreme linear detector in positive ion mode to determine the mass to charge ratio of the proteins. For analysis of the crystals by mass spectrometry, 2 crystals were washed in the initial buffer (20 mM HEPES pH 7.5 and 120 mM NaCl), then a 0.1% (v/v) acetonitrile solution. The crystals were dissolved in 15 μL of 20 mM HEPES pH 7.5 and 100 mM NaCl buffer and sent to the Biointerfaces Institute (McMaster University) for analysis.

### PilO bioinformatics analysis

The *P. aeruginosa* PAK PilO sequence was retrieved from the *Pseudomonas* Genome Database^44^ and was aligned using Jalview 2.8.2^45,46^ with a subset of Pseudomonads (*P. fluorescens*, *P. syringae* pv. phaseolicola 1448 A, *P. savastanoi, P. syringae, P. protegens* CHA0*, P. avellanae, P. stutzeri* A1501*, P. resinovorans, P. chlororaphis*). The mapped secondary structure elements are based on the new PilO_Δ109_ structure described herein. Generation of PilO mutants.

Sites for the introduction of single or double point mutations in PilO were chosen with reference to the new PilO_∆109_ structure (below). To maintain native stoichiometry and expression levels, mutations of interest were introduced onto the *P. aeruginosa* chromosome at the native *pilO* locus. First, codons for PilO residue substitutions were introduced into a pEX18Gm::*pilNOP* construct using the QuikChange Site-Directed Mutagenesis Kit (Stratagene) following the manufacturer’s protocol. Genes and mutations were sequenced (MOBIX) to verify their identity. Next, *pilO* mutants were constructed using a Flp-FRT (FLP recombination target) system as previously described^47^. Briefly, the suicide vectors containing the mutant *pilO* gene plus the flanking genes (*pilN* and *pilP*) to provide homologous regions for recombination were introduced into *E. coli* SM10 cells. The constructs were transferred by conjugation in a 1:9 ratio of *P. aeruginosa* to *E. coli*. The mixed culture was pelleted for 3 min at 2292 × *g* in a microcentrifuge, and the pellet was resuspended in 50 μL of LB, spot-plated on LB agar, and incubated overnight at 37 °C. A *P. aeruginosa* PAK strain which contained a *pilO::FRT* mutation^33^ was used as a recipient for the pEX18Gm::*pilNOP* constructs in mating experiments. After mating, cells were scraped from the LB agar plate, resuspended in 1 mL of LB and the *E. coli* SM10 donor was counterselected by plating on *Pseudomonas* isolation agar (PIA; Difco) containing Gm (100 μg/mL). Gm-resistant *P. aeruginosa* isolates were streaked on LB no salt plates with sucrose (1% (w/v) bacto-tryptone, 0.5% (w/v) bacto-yeast extract, 5% (w/v) sucrose) then incubated for 16 h at 30 °C. Select colonies were cultured in parallel on LB and LB plates supplemented with Gm. Gm-sensitive colonies were screened by PCR using *pilO* primers to confirm replacement of the FRT-disrupted gene, and PCR products of the expected size were sequenced to confirm incorporation of the desired mutations.

### Twitching motility assays

Twitching assays were performed as previously described^48^. Briefly, single colonies were stab inoculated to the bottom of a 1% LB agar plate. The plates were incubated for 36 h at 37 °C. Post incubation, the agar was carefully removed and the adherent bacteria stained with 1% (w/v) crystal violet dye, followed by washing with tap water to remove unbound dye. Areas of the twitching zones were measured using ImageJ software (NIH). All experiments were performed in triplicate with at least three independent replicates.

### Sheared surface protein preparation

Surface pili and flagella were analyzed as described previously^48^. Briefly, the strains of interest were streaked in a grid-like pattern on LB agar plates and incubated at 37 °C for ~16 h. The cells were scraped from the plates with glass coverslips and resuspended in 4.5 mL of PBS. Surface proteins were sheared by vortexing the cell suspensions for 30 s. Cells were transferred to three separate 1.5 mL Eppendorf tubes and pelleted by centrifugation at 11,688 × *g* for 5 min. Supernatant was transferred to fresh tubes and centrifuged at 11,688 × *g* for 20 min to remove remaining cells. Supernatants were transferred to new tubes and surface proteins were precipitated by adding 1/10 volume of 5 M NaCl and 30% (w/v) polyethylene glycol (PEG 8000, Sigma Aldrich) to each tube and incubating on ice for 90 min. Precipitated proteins were collected by centrifugation at 11,688 × *g*, resuspended in 150 μL of 1× SDS sample buffer (125 mM Tris, pH 6.8, 2% β-mercaptoethanol, 20% glycerol, 4% SDS and 0.001% bromophenol blue). Samples were boiled for 10 min and separated on 15% SDS-PAGE gels. Proteins were visualized by staining with Coomassie brilliant blue.

### Preparation of whole cell lysates

*P*. *aeruginosa* strains were grown on LB agar plates overnight at 37 °C. Cells were scraped from the surface and resuspended in 1 × PBS to an OD_600_ of 0.6. A 1 mL aliquot of cells was collected by centrifugation at 2292 × *g* for 3 min in a microcentrifuge. The cell pellet was resuspended in 100 μL of 1× SDS sample buffer and boiled for 10 min. Whole cell lysate samples were separated on 15% SDS-PAGE gels and subject to Western blot analysis.

### Western blot analysis

Whole cell lysate samples were separated on 15% SDS-PAGE gels and transferred to nitrocellulose membranes for 1 h at 225 mA. Membranes were blocked using a 5% (w/v) low fat skim milk powder in 1 × PBS for 1 h at room temperature on a shaking platform, followed by incubation with the appropriate antisera for 2 h at room temperature, at a dilutions as follows: PilM 1/2500, and PilNOP 1/1000 each. The membranes were washed twice in 1× PBS for 5 min then incubated in goat-anti-rabbit IgG-alkaline phosphatase conjugated secondary antibody (Bio-Rad) at a dilution of 1/3000 for 1 h at room temperature. The membranes were washed twice in 1× PBS for 5 min, and visualized with alkaline phosphatase developing reagent (Bio-Rad) following the manufacturer’s protocol.

### Bacterial Two-Hybrid Assay

Chemically competent *E. coli* BTH101 cells were co-transformed with various combinations of pUT18C and pKT25 protein fusions, and tested for interaction using MacConkey agar supplemented with activity using a 96-well plate-based assay as previously described^40^ with modifications. Briefly, BTH101 cells co-transformed with T18-PilO_Δ109_ and T25- PilO_Δ109_ fusion constructs with either the R169D or I170A mutations as indicated. Cells were grown at 30 °C in LB supplemented with Ap and Kn, shaking at 200 rpm overnight. LB broth containing antibiotics and 0.5 mM Isopropyl β-D-1-thiogalacto-pyranoside (IPTG; Sigma-Aldrich), were inoculated with a 1:5 dilution of an overnight culture and grown at 30 °C, 260 rpm shaking, to an optical density at 600 nm (OD_600_) of ~0.6 and standardized. Cells were spotted on MacConkey agar supplemented 1% maltose, and plates were incubated for 48 h at 30 °C.

## Electronic supplementary material

Supplementary Table S1
