# Supplementary material for: Conserved, unstructured regions in Pseudomonas aeruginosa PilO are important for type IVa pilus function
Source: Sci Rep. 2018 Feb 8;8:2600. doi: 10.1038/s41598-018-20925-w (PMC5805733; doi:10.1038/s41598-018-20925-w)
Supplement: Supplementary file 1 — Supplementary Table S1 [file 41598_2018_20925_MOESM1_ESM.docx]

**Supplementary Information for:**

**Conserved, unstructured regions in *Pseudomonas aeruginosa* PilO are important for
type IVa pilus function**

Leighton TL^1^, Mok MC^1,2^, Junop MS^2^, Howell PL^3,4,*^ and Burrows LL^1^* ^1^ Department of Biochemistry and Biomedical Sciences and the Michael G. DeGroote Institute for Infectious Disease Research, McMaster University, ON, CANADA.

^2^Department of Biochemistry, University of Western Ontario, ON, CANADA.
^3^Program in Molecular Structure & Function, The Hospital for Sick Children, ON, CANADA.
^4^Department of Biochemistry, University of Toronto, ON, CANADA.

Supplementary Table S1: Bacterial Strains & Vectors

| **Strain** | **Description** | **Source/ Reference** |
| --- | --- | --- |
| ***E. coli* strains** |  |  |
| DH5α | F– Φ80*lac*ZΔM15 Δ(*lac*ZYA-*arg*F) U169 *rec*A1 *end*A1 *hsd*R17 (rK–, mK+) *pho*A *sup*E44 λ– *thi*-1*gyr*A96 *rel*A1 | ThermoFisher Scientific |
| BL21 (DE3) | F– *omp*T *hsd*SB(rB–, mB–) *gal dcm*(DE3) | ThermoFisher Scientific |
| SM10 | thi-1, thr, leu, tonA, lacy, supE, recA, RP4-2-Tcr::Mu, Km^r^; mobilizes plasmids into *P. aeruginosa* via conjugation | ^1^ |
| BTH101 | F-, cya-99, araD139, galE15, galK16, rpsL1 (StrR), hsdR2, mcrA1, mcrB1, relA1 | Euromedex |
| ***P. aeruginosa* strains** |  |  |
| PAK | Wild-type | J. Boyd |
| *ΔpilM* | Deletion of *pilM* | ^2^ |
| *pilN::FRT* | FRT scar at position 124 within *pilN* | ^2^ |
| *pilO::FRT* | FRT scar at position 328 within *pilO* | ^2^ |
| *pilP::FRT* | FRT scar at position 86 within *pilP* | ^2^ |
| PilO LL132-133AA | *pilO* with L132A and L133A double point substitutions | This study |
| PilO PE134-135AL | *pilO* with P134A and E135L double point substitutions | This study |
| PilO F140A | *pilO* with F140A point substitution | This study |
| PilO Y141A | *pilO* with Y141A point substitution | This study |
| PilO L167A | *pilO* with L167A point substitution | This study |
| PilO P168A | *pilO* with P168A point substitution | This study |
| PilO R169D | *pilO* with R169D point substitution | This study |
| PilO I170A | *pilO* with I170A point substitution | This study |
| PilO TL172-173AA | *pilO* with T172A and L173A double point substitutions | This study |
| PilO H174A | *pilO* with H174A point substitution | This study |
| PilO D175R | *pilO* with D175R point substitution | This study |
| **Vectors** | Description | Source/ Reference |
| pEX18Gm | Suicide vector used for gene replacement, Gm^R^ | ^3^ |
| pEX18Gm::*pilNOP*  PilO LL132-133AA | Suicide vector containing *pilNOP* with *pilO* LL132-133AA, Gm^R^ | This study |
| pEX18Gm::*pilNOP*  PilO PE134-135AL | Suicide vector containing *pilNOP* with *pilO* PE134-135AL, Gm^R^ | This study |
| pEX18Gm::*pilNOP*  PilO F140A | Suicide vector containing *pilNOP* with *pilO* F140A, Gm^R^ | This study |
| pEX18Gm::*pilNOP*  PilO Y141A | Suicide vector containing *pilNOP* with *pilO* Y141A, Gm^R^ | This study |
| pEX18Gm::*pilNOP*  PilO L167A | Suicide vector containing *pilNOP* with *pilO* L167A, Gm^R^ | This study |
| pEX18Gm::*pilNOP*  PilO P168A | Suicide vector containing *pilNOP* with *pilO* P168A, Gm^R^ | This study |
| pEX18Gm::*pilNOP* PilO R169D | Suicide vector containing *pilNOP* with *pilO* R169D, Gm^R^ | This study |
| pEX18Gm::*pilNOP*  PilO I170A | Suicide vector containing *pilNOP* with *pilO* I170A, Gm^R^ | This study |
| pEX18Gm::*pilNOP*  PilO TL172-173AA | Suicide vector containing *pilNOP* with *pilO* TL172-173AA, Gm^R^ | This study |
| pEX18Gm::*pilNOP*  PilO H174A | Suicide vector containing *pilNOP* with *pilO* H174A, Gm^R^ | This study |
| pEX18Gm::*pilNOP*  PilO D175R | Suicide vector containing *pilNOP* with *pilO* D175R, Gm^R^ | This study |
| pEX18Gm::*pilNOP*  PilO F176A | Suicide vector containing *pilNOP* with *pilO* F176A, Gm^R^ | This study |
| pKT25 | Kn^R^ | ^4^ |
| pUT18C | Ap^R^ | ^4^ |
| pKT25::PilO_Δ109_ | Vector containing PilO_Δ109,_ Kn^R^ | This study |
| pUT18C::PilO_Δ109_ | Vector containing PilO_Δ109_, Ap^R^ | This study |
| pKT25::PilO_Δ109_ R169D | Vector containing PilO_Δ109_ with R169D substitution, Kn^R^ | This study |
| pUT18C::PilO_Δ109_ R169D | Vector containing PilO_Δ109_ with R169D substitution, Ap^R^ | This study |
| pKT25::PilO_Δ109_ I170A | Vector containing PilO_Δ109_ with I170A substitution, Kn^R^ | This study |
| pUT18C::PilO_Δ109_ I170A | Vector containing PilO_Δ109_ with I170A substitution, Ap^R^ | This study |

**Supplementary References:**

1 Simon, R., Priefer, U., Pulher, A. A broad host range mobilisation system for *in vivo* genetic engineering: transposon mutagenesis in Gram-negative bacteria. *Nat Biotech* **1**, 784-791 (1983).

2 Ayers, M. *et al.* PilM/N/O/P proteins form an inner membrane complex that affects the stability of the *Pseudomonas aeruginosa* type IV pilus secretin. *Journal of Molecular Biology* **394**, 128-142, doi:10.1016/j.jmb.2009.09.034 (2009).

3 Hoang, T. T., Karkhoff-Schweizer, R. R., Kutchma, A. J. & Schweizer, H. P. A broad-host-range Flp-FRT recombination system for site-specific excision of chromosomally-located DNA sequences: application for isolation of unmarked *Pseudomonas aeruginosa* mutants. *Gene* **212**, 77-86 (1998).

4 Karimova, G., Dautin, N. & Ladant, D. Interaction network among *Escherichia coli* membrane proteins involved in cell division as revealed by bacterial two-hybrid analysis. *Journal of Bacteriology* **187**, 2233-2243, doi:10.1128/JB.187.7.2233-2243.2005 (2005).

**Supplementary Figure S1. Uncropped gels and western blots used for Figure 5.**
